# Data solubility and parameters of adjustments (*α* and *β*) of phenanthrene in supercritical CO_2_ employing the modified Redlich–Kwong equation

**DOI:** 10.1016/j.dib.2018.10.038

**Published:** 2018-10-17

**Authors:** Fredy Colpas-Castillo, Arnulfo Tarón-Dunoyer, Rafael González-Cuello

**Affiliations:** a Department of Chemistry, Faculty of Exact and Natural Sciences, University of Cartagena, Cartagena. Colombia; b Department of Food Science, Faculty of Engineering, University of Cartagena, Cartagena, Colombia

## Abstract

This article contains data related to the research article entitled “Calculation method for determining Phenanthrene solubility in supercritical CO_2_ employing Redlich–Kwong modified equation” (Colpas et al., 2018) [1]. The presented data gives information on the physical properties of the solute and the solvent. The experimental solubilities of phenanthrene in equilibrium and those calculated using the modified Redlich–Kwong equation with the inclusion of the adjustment parameters *α* and *β* are shown, see Colpas et al. (2018) [1] and “Modified Redlich–Kwong equation of state for supercritical carbon dioxide” (Heidaryan and Jarrahian, 2013) [7]. The mean squared error (MSE) was calculated for the supercritical Phenanthrene–CO_2_ system at different temperatures above the critical point of the solvent.

## Specifications table

**Table:** 

Subject area	Chemical Engineering
More specific subject area	Thermodynamics/Equilibrium of solids in supercritical fluids
Type of data	Tables
How data was acquired	Employing the modified Redlich–Kwong equation, the non-linear simplex method to optimize the values, published literature.
Data format	Raw and Analyzed
Experimental factors	Phenanthrene’ solubilities in supercritical CO_2_ was calculated using the modified Redlich–Kwong equation including adjustable parameters such as alpha and beta (*α* and *β*). A comparison of the experimental data was made through an objective function optimizing alpha and beta values employing a simplex non-linear method.
Experimental features	Published data is used to calculate the solubilities of phenanthrene extracted with carbon dioxide under supercritical conditions at different temperature values.
Data source location	Universidad de Puerto Rico. Mayaguez. Puerto Rico.
Data accessibility	Data are available in this article
Related research article	Colpas, C.A., Tarón, D.A., and González, C.R. Calculation method for determining phenantrene solubility in supercritical CO_2_ employing Redlich–Kwong modified equation. Contemporary Engineering Sciences. 11 (40) (2018): 1971–1981.
	https://doi.org/10.12988/ces.2018.84180[1]

## Value of the data

• The calculated solubility data are useful for comparisons with those obtained using modified state equations with different adjustment parameters.
• The *α* and *β* parameters avoid the use of critical conditions of the solute, making relevant the solubility data calculated to apply to systems where the solute is a thermolabile substance.
• The average quadratic error (EQA) data indicate that the calculation method applies to other systems of interest for the pharmaceutical, food and chemical industry mainly.

## 1 Data

The data presented in this article include the experimental solubility of Phenanthrene (*Y*) at different temperatures and pressures in carbon dioxide under supercritical conditions [2]. Table 1. The physical properties of the solvent; molecular weight (*M*), critical pressure (*P_c_*), critical temperature (*T_c_*), acentric factor (*ω*), Table 2. The properties of the solute; molecular weight (*M*), *A* and *B* (variables in Redlich–Kwong equation of state) and molar volume (*V*^*sol*^), they were obtained from the literature [3], [4], [5]. The solubility values calculated using the modified Redlich–Kwong equation of state with adjustable parameters (α and *β*) are shown in Table 3.

**Table 1:** Experimental data of solubility in equilibrium of Phenanthrene in supercritical CO_2_.

***T* = 318** **K**		***T* = 328** **K**		***T* = 338** **K**	
***P*, (MPa)**	***Y* Molar fraction**	***P*, (MPa)**	***Y* Molar fraction**	***P*, (MPa)**	***Y* Molar fraction**
12	8.49 × 10^−4^	12	4.65 × 10^−4^	12	3.28 × 10^−4^
16	11.4^3^ × 10^−3^	16	1.51 × 10^−3^	16	1.18 × 10^−3^
20	1.70 × 10^−3^	20	2.14 × 10^−3^	20	2.37 × 10^−3^
24	2.23 × 10^−3^	24	2.79 × 10^−3^	24	3.28 × 10^−3^
28	2.28 × 10^−3^	28	3.19 × 10^−3^	28	3.84 × 10^−3^

**Table 2:** Physical properties of the solute.

**Solute**	***M* (g/mol)**	***P_c_* (MPa)**	***T_c_* (K)**	***ω***	***A***	***B* (k)**	**10**^**3**^**× *V****^**sol**^***(M**^**3**^**/mol)**	**Refs.**
Phenanthrene	178.24	3.17	882.55	0.3299	14.631	4873.4	0.1512	[3], [4], [5], [6]^a^

a *P_c_*, *T_c_* and *ω* from Refs. [3], [6]; *A* and *B* from Refs. [4], [6]; *V^sol^* from Refs. [5], [6].

**Table 3:** Solubility data calculated for the supercritical phenanthrene-CO_2_ system using the modified Redlich–Kwong equation of state.

**Supercritical temperature (K)**											
**323.15**				**328.15**				**338.15**			
*P*, (MPa)	Molar fraction	*α*	*β*	*P*, (MPa)	Molar fraction	*Α*	*β*	*P*, (MPa)	Molar fraction	*Α*	*β*
3.5 × 10^−7^	1	5.737	7.210	6.02 × 10^−7^	1	6.038	7.939	1.6 × 10^−6^	1	6.096	8.054
3.5 × 10^−6^	0.1	5.737	7.210	6.02 × 10^−6^	0.1	6.038	7.939	1.6 × 10^−5^	0.1	6.096	8.054
3.5 × 10^−5^	0.01	5.737	7.210	6.02 × 10^−5^	0.01	6.038	7.939	1.6 × 10^−4^	0.01	6.096	8.054
3.5 × 10^−4^	0.001	5.737	7.210	6.02 × 10^−4^	0.001	6.038	7.939	1.6 × 10^−3^	0.001	6.096	8.054
3.5 × 10^−3^	0.0001	5.737	7.210	6.02 × 10^−3^	0.0001	6.038	7.939	1.6 × 10^−2^	0.0001	6.096	8.054
3.5 × 10^−2^	1.02 × 10^−5^	5.737	7.210	6.02 × 10^−2^	1.03 × 10^−5^	6.038	7.939	1.6 × 10^−1^	1.09 × 10^−5^	6.096	8.054
0.3548	1.21 × 10^−6^	5.737	7.210	0.2	3.36 × 10^−6^	6.038	7.939	0.2	9.16 × 10^−6^	6.096	8.054
0.4	1.10 × 10^−6^	5.737	7.210	0.4	1.87 × 10^−6^	6.038	7.939	0.4	5.07 × 10^−6^	6.096	8.054
0.8	6.89 × 10^−7^	5.737	7.210	0.6	1.39 × 10^−6^	6.038	7.939	0.6	3.74 × 10^−6^	6.096	8.054
1.2	5.75 × 10^−7^	5.737	7.210	0.8	1.17 × 10^−6^	6.038	7.939	0.8	3.11 × 10^−6^	6.096	8.054
1.6	5.42 × 10^−7^	5.737	7.210	1	1.05 × 10^−6^	6.038	7.939	1	2.76 × 10^−6^	6.096	8.054
2	5.48 × 10^−7^	5.737	7.210	1.2	9.75 × 10^−7^	6.038	7.939	1.2	2.55 × 10^−6^	6.096	8.054
2.4	5.80 × 10^−7^	5.737	7.210	1.4	9.36 × 10^−7^	6.038	7.939	1.4	2.42 × 10^−6^	6.096	8.054
2.8	6.33 × 10^−7^	5.737	7.210	1.6	9.19 × 10^−7^	6.038	7.939	1.6	2.36 × 10^−6^	6.096	8.054
3.2	7.11 × 10^−7^	5.737	7.210	1.8	9.16 × 10^−7^	6.038	7.939	1.8	2.33 × 10^−6^	6.096	8.054
3.6	8.15 × 10^−7^	5.737	7.210	2	9.27 × 10^−7^	6.038	7.939	2	2.33 × 10^−6^	6.096	8.054
4	9.52 × 10^−7^	5.737	7.210	2.2	9.47 × 10^−7^	6.038	7.939	2.2	2.36 × 10^−6^	6.096	8.054
4.4	1.13 × 10^−6^	5.737	7.210	2.4	9.77 × 10^−7^	6.038	7.939	2.4	2.41 × 10^−6^	6.096	8.054
4.8	1.37 × 10^−6^	5.737	7.210	2.6	1.02 × 10^−6^	6.038	7.939	2.6	2.48 × 10^−6^	6.096	8.054
5.2	1.68 × 10^−6^	5.737	7.210	2.8	1.07 × 10^−6^	6.038	7.939	2.8	2.56 × 10^−6^	6.096	8.054
5.6	2.09 × 10^−6^	5.737	7.210	3	1.12 × 10^−6^	6.038	7.939	3	2.67 × 10^−6^	6.096	8.054
6	2.65 ×10^−6^	5.737	7.210	3.2	1.19 × 10^−6^	6.038	7.939	3.2	2.80 × 10^−6^	6.096	8.054
6.4	3.41 × 10^−6^	5.737	7.210	3.4	1.27 × 10^−6^	6.038	7.939	3.4	2.94 × 10^−6^	6.096	8.054
6.8	4.48 × 10^−6^	5.737	7.210	3.6	1.36 × 10^−6^	6.038	7.939	3.6	3.11 × 10^−6^	6.096	8.054
7.2	5.99 × 10^−6^	5.737	7.210	3.8	1.46 ×1 0^−6^	6.038	7.939	3.8	3.30 × 10^−6^	6.096	8.054
7.6	8.20 × 10^−6^	5.737	7.210	4	1.58 × 10^−6^	6.038	7.939	4	3.52 × 10^−6^	6.096	8.054
8	1.15 × 10^−5^	5.737	7.210	4.2	1.71 × 10^−6^	6.038	7.939	4.2	3.76 × 10^−6^	6.096	8.054
8.4	166 × 10^−5^	5.737	7.210	4.4	1.86 × 10^−6^	6.038	7.939	4.4	4.03 × 10^−6^	6.096	8.054
8.8	2.48 × 10^−5^	5.737	7.210	4.6	2.03 × 10^−6^	6.038	7.939	4.6	4.33 × 10^−6^	6.096	8.054
9.2	3.80 × 10^−5^	5.737	7.210	4.8	2.23 × 10^−6^	6.038	7.939	4.8	4.68 × 10^−6^	6.096	8.054
9.6	5.98 × 10^−5^	5.737	7.210	5	2.45 × 10^−6^	6.038	7.939	5	5.06 × 10^−6^	6.096	8.054
10	9.46 × 10^−5^	5.737	7.210	5.2	2.70 × 10^−6^	6.038	7.939	5.2	5.49 × 10^−6^	6.096	8.054
10.4	0.000145	5.737	7.210	5.4	2.99 × 10^−6^	6.038	7.939	5.4	5.97 × 10^−6^	6.096	8.054
10.43	0.000150	5.737	7.210	5.6	3.33 × 10^−6^	6.038	7.939	5.6	6.50 × 10^−6^	6.096	8.054
10.8	0.000213	5.737	7.210	5.8	3.71 × 10^−6^	6.038	7.939	5.8	7.11 × 10^−6^	6.096	8.054
11.2	0.000293	5.737	7.210	6	4.15 × 10^−6^	6.038	7.939	6	7.79 × 10^−6^	6.096	8.054
11.6	0.000381	5.737	7.210	6.2	4.66 × 10^−6^	6.038	7.939	6.2	8.55 × 10^−6^	6.096	8.054
11.81	0.000429	5.737	7.210	6.4	5.24 × 10^−6^	6.038	7.939	6.4	9.41 × 10^−6^	6.096	8.054
12	0.000472	5.737	7.210	6.6	5.93 × 10^−6^	6.038	7.939	6.6	1.04 × 10^−5^	6.096	8.054
12.4	0.000564	5.737	7.210	6.8	6.72 × 10^−6^	6.038	7.939	6.8	1.15 × 10^−5^	6.096	8.054
12.8	0.000656	5.737	7.210	7	7.65 × 10^−6^	6.038	7.939	7	1.27 × 10^−5^	6.096	8.054
13.2	0.000746	5.737	7.210	7.2	8.74 × 10^−6^	6.038	7.939	7.2	1.41 × 10^−5^	6.096	8.054
13.6	0.000834	5.737	7.210	7.4	1.00 × 10^−5^	6.038	7.939	7.4	1.57 × 10^−5^	6.096	8.054
13.88	0.000895	5.737	7.210	7.6	1.15 × 10^−5^	6.038	7.939	7.6	1.75 × 10^−5^	6.096	8.054
14	0.000920	5.737	7.210	7.8	1.34 × 10^−5^	6.038	7.939	7.8	1.96 × 10^−5^	6.096	8.054
14.4	0.001003	5.737	7.210	8	1.55 × 10^−5^	6.038	7.939	8	2.19 × 10^−5^	6.096	8.054
14.8	0.001083	5.737	7.210	8.2	1.81 × 10^−5^	6.038	7.939	8.2	2.46 × 10^−5^	6.096	8.054
15.2	0.001161	5.737	7.210	8.4	2.12 × 10^−5^	6.038	7.939	8.4	2.77 × 10^−5^	6.096	8.054
15.6	0.001236	5.737	7.210	8.6	2.49 × 10^−5^	6.038	7.939	8.6	3.12 × 10^−5^	6.096	8.054
16	0.001308	5.737	7.210	8.8	2.94 × 10^−5^	6.038	7.939	8.8	3.52 × 10^−5^	6.096	8.054
16.4	0.001377	5.737	7.210	9	3.49 × 10^−5^	6.038	7.939	9	3.98 × 10^−5^	6.096	8.054
16.8	0.001444	5.737	7.210	9.2	4.16 × 10^−5^	6.038	7.939	9.2	4.51 × 10^−5^	6.096	8.054
17.2	0.001508	5.737	7.210	9.4	4.97 × 10^−5^	6.038	7.939	9.4	5.12 × 10^−5^	6.096	8.054
17.6	0.001570	5.737	7.210	9.6	5.97 × 10^−5^	6.038	7.939	9.6	5.82 × 10^−5^	6.096	8.054
18	0.001629	5.737	7.210	9.8	7.18 × 10^−5^	6.038	7.939	9.8	6.62 × 10^−5^	6.096	8.054
18.4	0.00168	5.737	7.210	10	8.64 × 10^−5^	6.038	7.939	10	7.54 × 10^−5^	6.096	8.054
18.8	0.00174	5.737	7.210	10.2	2.03 × 10^−6^	6.038	7.939	10.2	8.60 × 10^−5^	6.096	8.054
19.2	0.00179	5.737	7.210	10.4	2.23 × 10^−6^	6.038	7.939	10.4	9.81 × 10^−5^	6.096	8.054
19.6	0.00184	5.737	7.210	10.6	0.0001502	6.038	7.939	10.43	0.0001505	6.096	8.054
20	0.00189	5.737	7.210	10.8	0.0001792	6.038	7.939	10.6	0.0001118	6.096	8.054
20.4	0.00194	5.737	7.210	11	0.0002125	6.038	7.939	10.8	0.0001276	6.096	8.054
20.8	0.00198	5.737	7.210	11.2	0.0002500	6.038	7.939	11	0.0001454	6.096	8.054
21.2	0.00202	5.737	7.210	11.4	0.0002914	6.038	7.939	11.2	0.0001656	6.096	8.054
21.6	0.00206	5.737	7.210	11.6	0.0003365	6.038	7.939	11.4	0.0001883	6.096	8.054
22	0.00210	5.737	7.210	11.8	0.0003849	6.038	7.939	11.6	0.0002136	6.096	8.054
22.4	0.00214	5.737	7.210	12	0.0004360	6.038	7.939	11.8	0.0002418	6.096	8.054
22.8	0.00218	5.737	7.210	12.2	0.0004893	6.038	7.939	12	0.0002729	6.096	8.054
23.2	0.00221	5.737	7.210	12.4	0.0005440	6.038	7.939	12	0.0002728	6.096	8.054
23.6	0.00225	5.737	7.210	12.6	0.0006010	6.038	7.939	12.2	0.0003068	6.096	8.054
24	0.00222	5.737	7.210	12.8	0.0006584	6.038	7.939	12.4	0.0003438	6.096	8.054
24.4	0.00231	5.737	7.210	13	0.0007165	6.038	7.939	12.6	0.0003837	6.096	8.054
24.8	0.00234	5.737	7.210	13.2	0.0007749	6.038	7.939	12,.8	0.0004265	6.096	8.054
25.2	0.00237	5.737	7.210	13.4	0.0008334	6.038	7.939	13	0.0004720	6.096	8.054
25.6	0.00239	5.737	7.210	13.6	0.0008918	6.038	7.939	13.2	0.0005200	6.096	8.054
26	0.00242	5.737	7.210	13.8	0.0009498	6.038	7.939	13.4	0.0005704	6.096	8.054
26.4	0.00244	5.737	7.210	14	0.0010074	6.038	7.939	13.6	0.0006229	6.096	8.054
26.8	0.00247	5.737	7.210	14.2	0.0010644	6.038	7.939	13.8	0.0006774	6.096	8.054
27.2	0.00249	5.737	7.210	14.4	0.0011207	6.038	7.939	14	0.0007336	6.096	8.054
27.6	0.00251	5.737	7.210	14.6	0.0011763	6.038	7.939	14.2	0.0007912	6.096	8.054
27.67	0.00252	5.737	7.210	14.8	0.0012310	6.038	7.939	14.4	0.0008501	6.096	8.054
28	0.00253	5.737	7.210	15	0.0012849	6.038	7.939	14.6	0.0009100	6.096	8.054
28.4	0.00255	5.737	7.210	15.2	0.0013379	6.038	7.939	14.8	0.0009707	6.096	8.054
28.8	0.00257	5.737	7.210	15.4	0.0013899	6.038	7.939	15	0.0010320	6.096	8.054
29.2	0.00259	5.737	7.210	15.6	0.0014409	6.038	7.939	15.2	0.0010938	6.096	8.054
29.6	0.00260	5.737	7.210	15.8	0.0014910	6.038	7.939	15.4	0.0011558	6.096	8.054
30	0.00262	5.737	7.210	16	0.0015400	6.038	7.939	15.6	0.0012180	6.096	8.054

## 2 Experimental design, materials and methods

The solubility data are determined by employing the modified Redlich–Kwong equation of state. The modification of the equation was made from the inclusion of adjustable parameters called alpha and beta (*α* and *β*), which are relevant because when they are used it is not necessary to know the solute critical conditions. The solubilities values (calculated and experimental) are compared by an objective function which is optimized by applying the non-linear simplex method.

### 2.1 Mathematical details

The basic equation used to calculate the solubility of solids with low vapor pressure in supercritical fluids can be expressed as:

(1) $$y_{2}=\frac{P_{2}^{sub}}{P{\hat{\phi }}_{2}^{V}}\exp\left(\frac{V_{2}^{sol}P)}{RT}\right)$$

where *y*_*2*_ is the solubility of solids with low vapor pressure in supercritical fluids, the fugacity coefficient, *P*^*sat*^ the saturation pressure, *V*^*sol*^ is the solid molar volume of the solute, *R* the universal constant of the gases, *T* the temperature and P the system pressure. Subscript 2 refers to the solid component. All terms can be obtained experimentally except ${\hat{\phi }}_{2}^{V}$, The Redlich–Kwong equation is used to calculate the fugacity coefficient, the expression for the calculation is as follows:

(2) $$\ln\;{\hat{\phi }}_{2}=\left(Z-1\right)\frac{b_{2}}{b_{1}}-\;\ln\left(Z-B\right)+\frac{a}{bRT^{1.5}}\left[\frac{2\left(y_{1}a_{12}+y_{2}a_{2}\right)}{a}-\frac{b_{2}}{b}\right]\ln\;\frac{Z+B}{Z}$$

where *A* and *B* are the adjustable parameters, *a*_1_ and *b*_1_ are the solute Van der Waals parameters of solute, *a*_2_ and *b*_2_ are the solvent Van der Waals parameters, *a* and *b* are the mixture parameters and *Z* is the compressibility factor, which depend on the solubility. For the fluid phase, *a* and *b* were calculated employing Van der Waals mixing rule, it can be observed that solubility is a *Z* function.

To infinite dilution la Eq. (2) can be rewritten as follows:

(3) $$\ln\;{\hat{\phi }}_{2}^{\infty }=\left(Z-1\right)\frac{b_{2}}{b_{1}}-\;\ln\left(Z-B\right)+\frac{A}{B}\left(2\sqrt{\frac{a_{2}}{a_{1}}}-\frac{b_{2}}{b_{1}}\right)\ln\;\frac{Z+B}{Z}$$

The final equation for the fugacity coefficient at infinite dilution is:

(4) $$\ln\;{\hat{\phi }}_{2}^{\infty }=\beta \left(Z-1\right)-\;\ln\left(Z-B\right)+\frac{A}{B}\left(\beta -2\alpha \right)\ln\;\frac{Z+B}{Z}$$

where *α* is the adjustment parameter with respect to the molecular interactions between the solute and the solvent, *β* the adjustment parameter in relation to the molecular size between the solute and the solvent.

The mean squared error was calculated through the following equation

(5) $$\mathrm{ECM}=100\sqrt{\frac{\sum_{i=1}^{N_{p}}{\left(y_{i,\mathrm{cal}}-y_{i,\exp}\right)}^{2}}{N_{p}}}$$

where *y*_*i,cal*_ is the solubility calculated from component *i*, *y*_*i,exp*_ the experimental solubility of component *i* and *N*_*p*_ is the data number.

The reader can find further details in our previous paper [1], and another manuscript [2].
